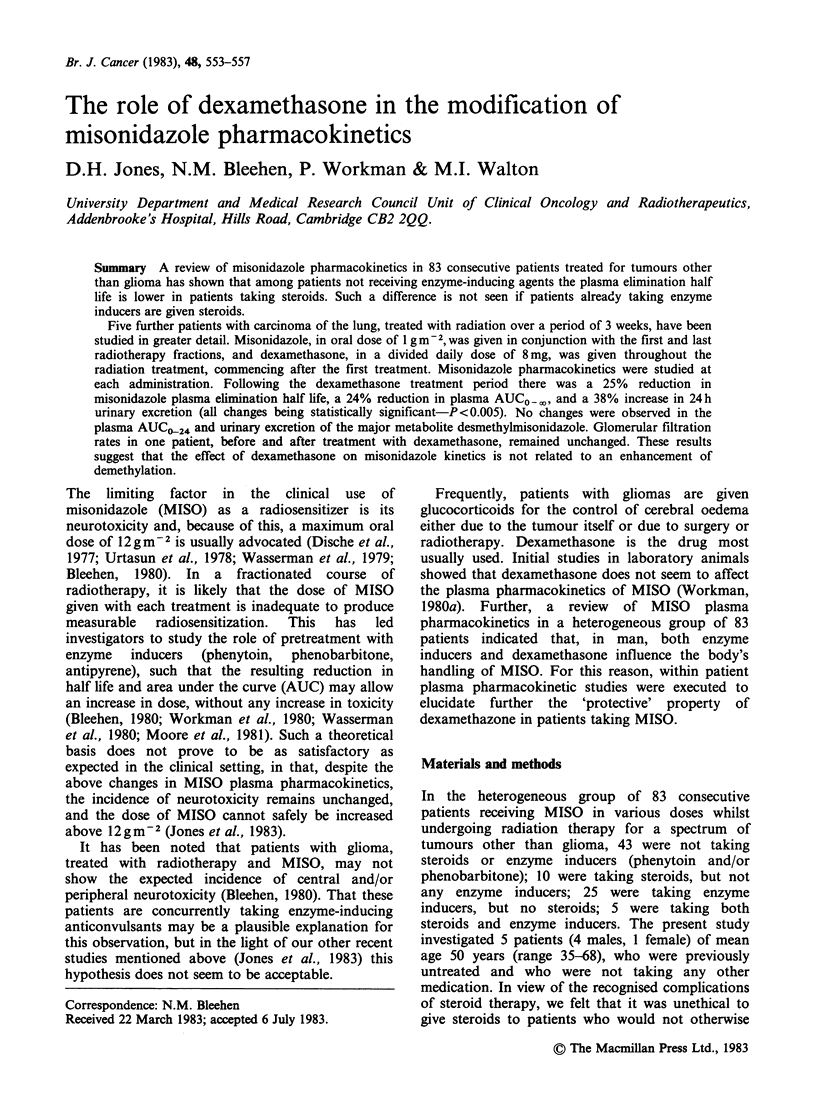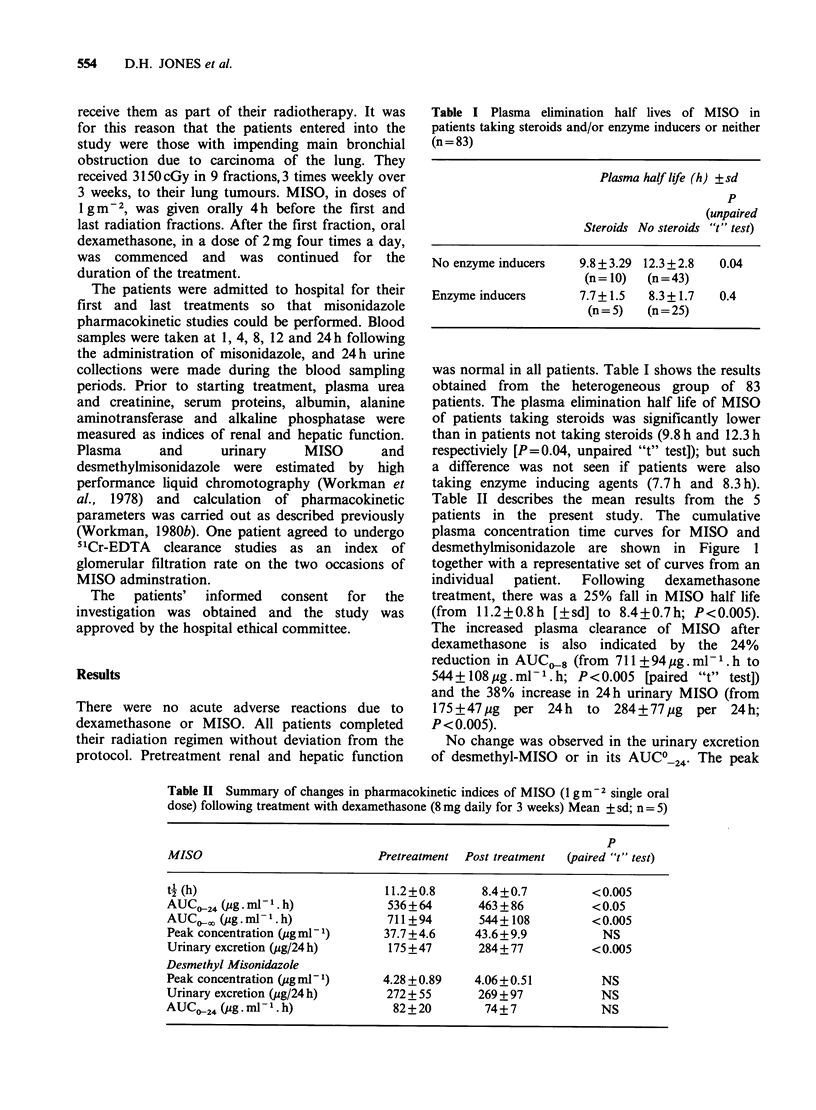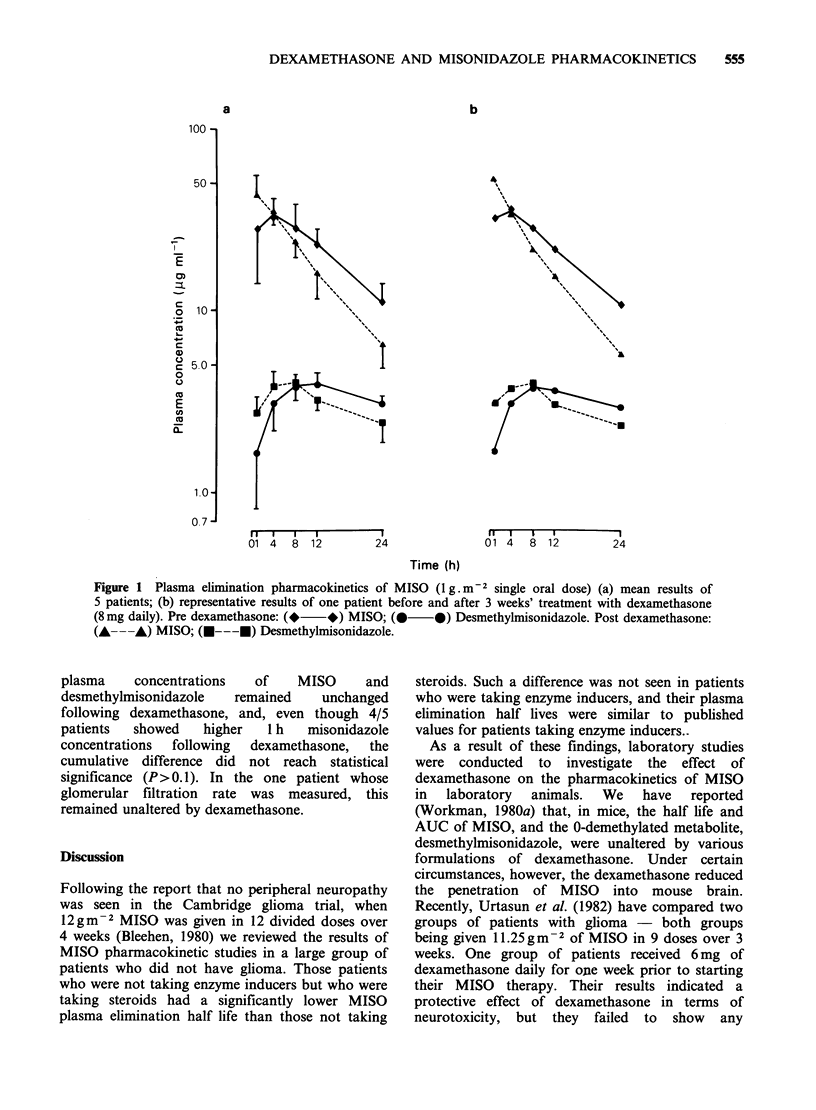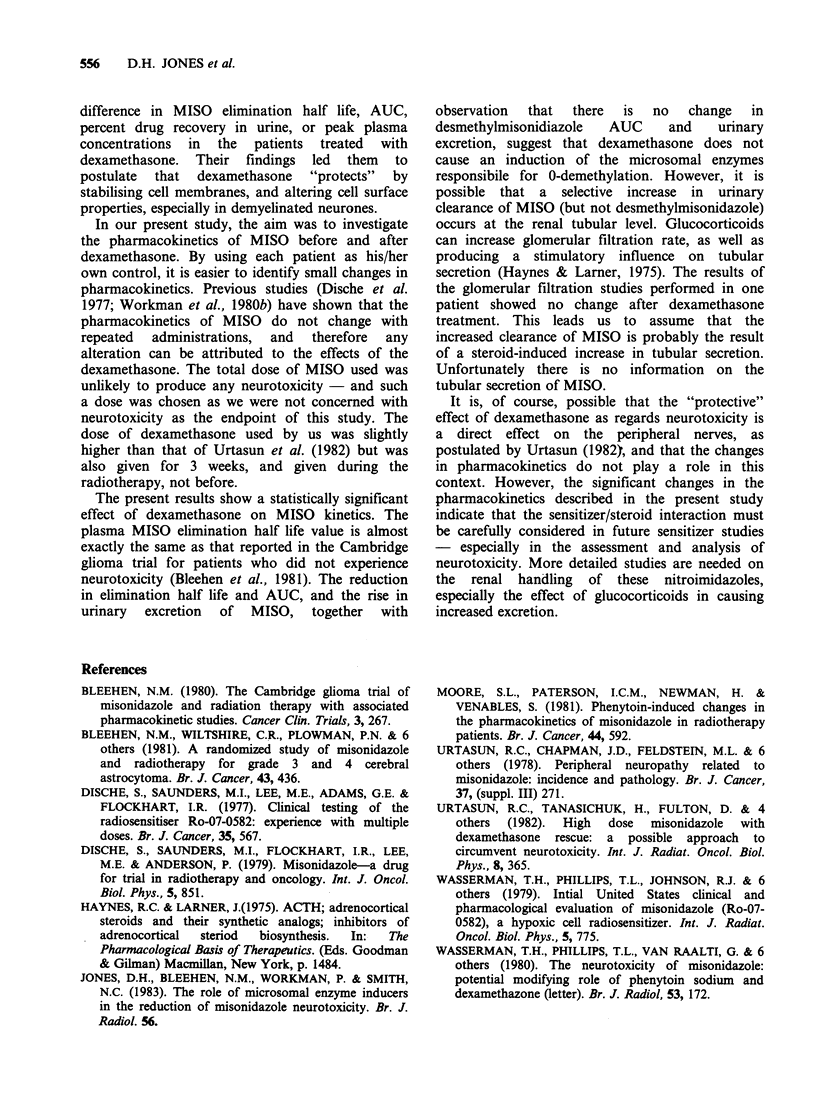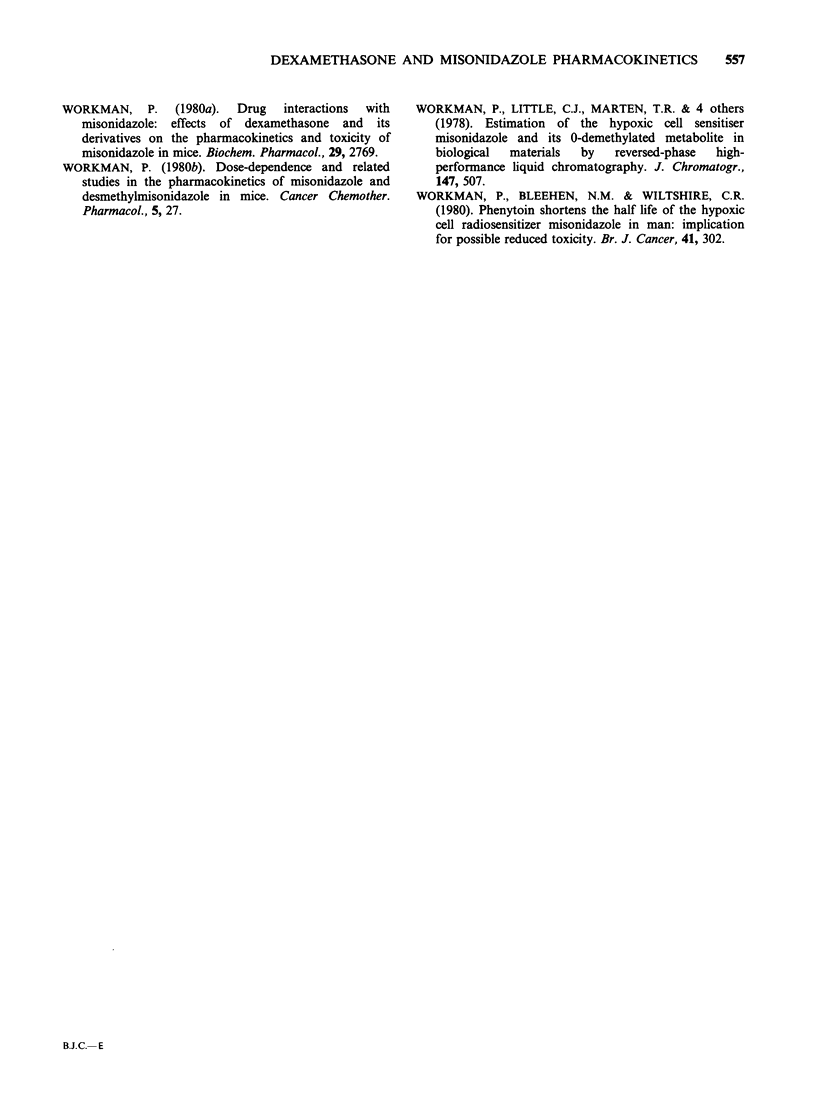# The role of dexamethasone in the modification of misonidazole pharmacokinetics.

**DOI:** 10.1038/bjc.1983.228

**Published:** 1983-10

**Authors:** D. H. Jones, N. M. Bleehen, P. Workman, M. I. Walton

## Abstract

A review of misonidazole pharmacokinetics in 83 consecutive patients treated for tumours other than glioma has shown that among patients not receiving enzyme-inducing agents the plasma elimination half life is lower in patients taking steroids. Such a difference is not seen if patients already taking enzyme inducers are given steroids. Five further patients with carcinoma of the lung, treated with radiation over a period of 3 weeks, have been studied in greater detail. Misonidazole, in oral dose of 1 gm-2, was given in conjunction with the first and last radiotherapy fractions, and dexamethasone, in a divided daily dose of 8 mg, was given throughout the radiation treatment, commencing after the first treatment. Misonidazole pharmacokinetics were studied at each administration. Following the dexamethasone treatment period there was a 25% reduction in misonidazole plasma elimination half life, a 24% reduction in plasma AUC0-00, and a 38% increase in 24 h urinary excretion (all changes being statistically significant--P less than 0.005). No changes were observed in the plasma AUC0-24 and urinary excretion of the major metabolite desmethylmisonidazole. Glomerular filtration rates in one patient, before and after treatment with dexamethasone, remained unchanged. These results suggest that the effect of dexamethasone on misonidazole kinetics is not related to an enhancement of demethylation.


Br. J. Cancer (1983), 48, 553–557

### The role of dexamethasone in the modification of misonidazole pharmacokinetics

D.H. Jones, N.M. Bleehen, P. Workman & M.I. Walton

*University Department and Medical Research Council Unit of Clinical Oncology and Radiotherapeutics, Addenbrooke's Hospital, Hills Road, Cambridge CB2 2QQ.*

**Summary** A review of misonidazole pharmacokinetics in 83 consecutive patients treated for tumours other than glioma has shown that among patients not receiving enzyme-inducing agents the plasma elimination half life is lower in patients taking steroids. Such a difference is not seen if patients already taking enzyme inducers are given steroids.

Five further patients with carcinoma of the lung, treated with radiation over a period of 3 weeks, have been studied in greater detail. Misonidazole, in oral dose of $1\,gm^{-2}$, was given in conjunction with the first and last radiotherapy fractions, and dexamethasone, in a divided daily dose of 8 mg, was given throughout the radiation treatment, commencing after the first treatment. Misonidazole pharmacokinetics were studied at each administration. Following the dexamethasone treatment period there was a 25% reduction in misonidazole plasma elimination half life, a 24% reduction in plasma $AUC_{0-\infty}$, and a 38% increase in 24 h urinary excretion (all changes being statistically significant—$P<0.005$). No changes were observed in the plasma $AUC_{0-24}$ and urinary excretion of the major metabolite desmethylmisonidazole. Glomerular filtration rates in one patient, before and after treatment with dexamethasone, remained unchanged. These results suggest that the effect of dexamethasone on misonidazole kinetics is not related to an enhancement of demethylation.

The limiting factor in the clinical use of misonidazole (MISO) as a radiosensitizer is its neurotoxicity and, because of this, a maximum oral dose of $12\,gm^{-2}$ is usually advocated (Dische *et al.*, 1977; Urtasun *et al.*, 1978; Wasserman *et al.*, 1979; Bleehen, 1980). In a fractionated course of radiotherapy, it is likely that the dose of MISO given with each treatment is inadequate to produce measurable radiosensitization. This has led investigators to study the role of pretreatment with enzyme inducers (phenytoin, phenobarbitone, antipyrene), such that the resulting reduction in half life and area under the curve (AUC) may allow an increase in dose, without any increase in toxicity (Bleehen, 1980; Workman *et al.*, 1980; Wasserman *et al.*, 1980; Moore *et al.*, 1981). Such a theoretical basis does not prove to be as satisfactory as expected in the clinical setting, in that, despite the above changes in MISO plasma pharmacokinetics, the incidence of neurotoxicity remains unchanged, and the dose of MISO cannot safely be increased above $12\,gm^{-2}$ (Jones *et al.*, 1983).

It has been noted that patients with glioma, treated with radiotherapy and MISO, may not show the expected incidence of central and/or peripheral neurotoxicity (Bleehen, 1980). That these patients are concurrently taking enzyme-inducing anticonvulsants may be a plausible explanation for this observation, but in the light of our other recent studies mentioned above (Jones *et al.*, 1983) this hypothesis does not seem to be acceptable.

Correspondence: N.M. Bleehen
Received 22 March 1983; accepted 6 July 1983.

Frequently, patients with gliomas are given glucocorticoids for the control of cerebral oedema either due to the tumour itself or due to surgery or radiotherapy. Dexamethasone is the drug most usually used. Initial studies in laboratory animals showed that dexamethasone does not seem to affect the plasma pharmacokinetics of MISO (Workman, 1980*a*). Further, a review of MISO plasma pharmacokinetics in a heterogeneous group of 83 patients indicated that, in man, both enzyme inducers and dexamethasone influence the body's handling of MISO. For this reason, within patient plasma pharmacokinetic studies were executed to elucidate further the 'protective' property of dexamethazone in patients taking MISO.

### Materials and methods

In the heterogeneous group of 83 consecutive patients receiving MISO in various doses whilst undergoing radiation therapy for a spectrum of tumours other than glioma, 43 were not taking steroids or enzyme inducers (phenytoin and/or phenobarbitone); 10 were taking steroids, but not any enzyme inducers; 25 were taking enzyme inducers, but no steroids; 5 were taking both steroids and enzyme inducers. The present study investigated 5 patients (4 males, 1 female) of mean age 50 years (range 35–68), who were previously untreated and who were not taking any other medication. In view of the recognised complications of steroid therapy, we felt that it was unethical to give steroids to patients who would not otherwise

© The Macmillan Press Ltd., 1983

receive them as part of their radiotherapy. It was for this reason that the patients entered into the study were those with impending main bronchial obstruction due to carcinoma of the lung. They received 3150 cGy in 9 fractions, 3 times weekly over 3 weeks, to their lung tumours. MISO, in doses of $1\,gm^{-2}$, was given orally 4h before the first and last radiation fractions. After the first fraction, oral dexamethasone, in a dose of 2 mg four times a day, was commenced and was continued for the duration of the treatment.

The patients were admitted to hospital for their first and last treatments so that misonidazole pharmacokinetic studies could be performed. Blood samples were taken at 1, 4, 8, 12 and 24 h following the administration of misonidazole, and 24 h urine collections were made during the blood sampling periods. Prior to starting treatment, plasma urea and creatinine, serum proteins, albumin, alanine aminotransferase and alkaline phosphatase were measured as indices of renal and hepatic function. Plasma and urinary MISO and desmethylmisonidazole were estimated by high performance liquid chromotography (Workman *et al.*, 1978) and calculation of pharmacokinetic parameters was carried out as described previously (Workman, 1980*b*). One patient agreed to undergo $^{51}$Cr-EDTA clearance studies as an index of glomerular filtration rate on the two occasions of MISO adminstration.

The patients' informed consent for the investigation was obtained and the study was approved by the hospital ethical committee.

### Results

There were no acute adverse reactions due to dexamethasone or MISO. All patients completed their radiation regimen without deviation from the protocol. Pretreatment renal and hepatic function was normal in all patients. Table I shows the results obtained from the heterogeneous group of 83 patients. The plasma elimination half life of MISO of patients taking steroids was significantly lower than in patients not taking steroids (9.8 h and 12.3 h respectiviely [$P=0.04$, unpaired "t" test]); but such a difference was not seen if patients were also taking enzyme inducing agents (7.7 h and 8.3 h). Table II describes the mean results from the 5 patients in the present study. The cumulative plasma concentration time curves for MISO and desmethylmisonidazole are shown in Figure 1 together with a representative set of curves from an individual patient. Following dexamethasone treatment, there was a 25% fall in MISO half life (from $11.2 \pm 0.8$ h [$\pm$sd] to $8.4 \pm 0.7$ h; $P<0.005$). The increased plasma clearance of MISO after dexamethasone is also indicated by the 24% reduction in $AUC_{0-8}$ (from $711 \pm 94\,\mu g.ml^{-1}.h$ to $544 \pm 108\,\mu g.ml^{-1}.h$; $P<0.005$ [paired "t" test]) and the 38% increase in 24 h urinary MISO (from $175 \pm 47\,\mu g$ per 24 h to $284 \pm 77\,\mu g$ per 24 h; $P<0.005$).

No change was observed in the urinary excretion of desmethyl-MISO or in its $AUC^{0}_{-24}$. The peak

**Table I** Plasma elimination half lives of MISO in patients taking steroids and/or enzyme inducers or neither (n=83)

| | *Plasma half life (h)* $\pm$*sd* | | |
|---|---|---|---|
| | *Steroids* | *No steroids* | *P (unpaired "t" test)* |
| No enzyme inducers | 9.8±3.29 (n=10) | 12.3±2.8 (n=43) | 0.04 |
| Enzyme inducers | 7.7±1.5 (n=5) | 8.3±1.7 (n=25) | 0.4 |

**Table II** Summary of changes in pharmacokinetic indices of MISO ($1\,gm^{-2}$ single oral dose) following treatment with dexamethasone (8 mg daily for 3 weeks) Mean ±sd; n=5)

| *MISO* | *Pretreatment* | *Post treatment* | *P (paired "t" test)* |
|---|---|---|---|
| $t_{\frac{1}{2}}$ (h) | 11.2±0.8 | 8.4±0.7 | <0.005 |
| $AUC_{0-24}$ ($\mu g.ml^{-1}.h$) | 536±64 | 463±86 | <0.05 |
| $AUC_{0-\infty}$ ($\mu g.ml^{-1}.h$) | 711±94 | 544±108 | <0.005 |
| Peak concentration ($\mu g\,ml^{-1}$) | 37.7±4.6 | 43.6±9.9 | NS |
| Urinary excretion ($\mu g$/24 h) | 175±47 | 284±77 | <0.005 |
| *Desmethyl Misonidazole* | | | |
| Peak concentration ($\mu g\,ml^{-1}$) | 4.28±0.89 | 4.06±0.51 | NS |
| Urinary excretion ($\mu g$/24 h) | 272±55 | 269±97 | NS |
| $AUC_{0-24}$ ($\mu g.ml^{-1}.h$) | 82±20 | 74±7 | NS |

**Figure 1** Plasma elimination pharmacokinetics of MISO ($1\,g.m^{-2}$ single oral dose) (a) mean results of 5 patients; (b) representative results of one patient before and after 3 weeks' treatment with dexamethasone (8 mg daily). Pre dexamethasone: (◆——◆) MISO; (●——●) Desmethylmisonidazole. Post dexamethasone: (▲---▲) MISO; (■---■) Desmethylmisonidazole.

plasma concentrations of MISO and desmethylmisonidazole remained unchanged following dexamethasone, and, even though 4/5 patients showed higher 1 h misonidazole concentrations following dexamethasone, the cumulative difference did not reach statistical significance ($P > 0.1$). In the one patient whose glomerular filtration rate was measured, this remained unaltered by dexamethasone.

### Discussion

Following the report that no peripheral neuropathy was seen in the Cambridge glioma trial, when $12\,gm^{-2}$ MISO was given in 12 divided doses over 4 weeks (Bleehen, 1980) we reviewed the results of MISO pharmacokinetic studies in a large group of patients who did not have glioma. Those patients who were not taking enzyme inducers but who were taking steroids had a significantly lower MISO plasma elimination half life than those not taking steroids. Such a difference was not seen in patients who were taking enzyme inducers, and their plasma elimination half lives were similar to published values for patients taking enzyme inducers..

As a result of these findings, laboratory studies were conducted to investigate the effect of dexamethasone on the pharmacokinetics of MISO in laboratory animals. We have reported (Workman, 1980*a*) that, in mice, the half life and AUC of MISO, and the 0-demethylated metabolite, desmethylmisonidazole, were unaltered by various formulations of dexamethasone. Under certain circumstances, however, the dexamethasone reduced the penetration of MISO into mouse brain. Recently, Urtasun *et al.* (1982) have compared two groups of patients with glioma — both groups being given $11.25\,gm^{-2}$ of MISO in 9 doses over 3 weeks. One group of patients received 6 mg of dexamethasone daily for one week prior to starting their MISO therapy. Their results indicated a protective effect of dexamethasone in terms of neurotoxicity, but they failed to show any difference in MISO elimination half life, AUC, percent drug recovery in urine, or peak plasma concentrations in the patients treated with dexamethasone. Their findings led them to postulate that dexamethasone "protects" by stabilising cell membranes, and altering cell surface properties, especially in demyelinated neurones.

In our present study, the aim was to investigate the pharmacokinetics of MISO before and after dexamethasone. By using each patient as his/her own control, it is easier to identify small changes in pharmacokinetics. Previous studies (Dische *et al.* 1977; Workman *et al.*, 1980*b*) have shown that the pharmacokinetics of MISO do not change with repeated administrations, and therefore any alteration can be attributed to the effects of the dexamethasone. The total dose of MISO used was unlikely to produce any neurotoxicity — and such a dose was chosen as we were not concerned with neurotoxicity as the endpoint of this study. The dose of dexamethasone used by us was slightly higher than that of Urtasun *et al.* (1982) but was also given for 3 weeks, and given during the radiotherapy, not before.

The present results show a statistically significant effect of dexamethasone on MISO kinetics. The plasma MISO elimination half life value is almost exactly the same as that reported in the Cambridge glioma trial for patients who did not experience neurotoxicity (Bleehen *et al.*, 1981). The reduction in elimination half life and AUC, and the rise in urinary excretion of MISO, together with observation that there is no change in desmethylmisonidiazole AUC and urinary excretion, suggest that dexamethasone does not cause an induction of the microsomal enzymes responsibile for 0-demethylation. However, it is possible that a selective increase in urinary clearance of MISO (but not desmethylmisonidazole) occurs at the renal tubular level. Glucocorticoids can increase glomerular filtration rate, as well as producing a stimulatory influence on tubular secretion (Haynes & Larner, 1975). The results of the glomerular filtration studies performed in one patient showed no change after dexamethasone treatment. This leads us to assume that the increased clearance of MISO is probably the result of a steroid-induced increase in tubular secretion. Unfortunately there is no information on the tubular secretion of MISO.

It is, of course, possible that the "protective" effect of dexamethasone as regards neurotoxicity is a direct effect on the peripheral nerves, as postulated by Urtasun (1982), and that the changes in pharmacokinetics do not play a role in this context. However, the significant changes in the pharmacokinetics described in the present study indicate that the sensitizer/steroid interaction must be carefully considered in future sensitizer studies — especially in the assessment and analysis of neurotoxicity. More detailed studies are needed on the renal handling of these nitroimidazoles, especially the effect of glucocorticoids in causing increased excretion.

### References

BLEEHEN, N.M. (1980). The Cambridge glioma trial of misonidazole and radiation therapy with associated pharmacokinetic studies. *Cancer Clin. Trials*, **3**, 267.

BLEEHEN, N.M., WILTSHIRE, C.R., PLOWMAN, P.N. & 6 others (1981). A randomized study of misonidazole and radiotherapy for grade 3 and 4 cerebral astrocytoma. *Br. J. Cancer*, **43**, 436.

DISCHE, S., SAUNDERS, M.I., LEE, M.E., ADAMS, G.E. & FLOCKHART, I.R. (1977). Clinical testing of the radiosensitiser Ro-07-0582: experience with multiple doses. *Br. J. Cancer*, **35**, 567.

DISCHE, S., SAUNDERS, M.I., FLOCKHART, I.R., LEE, M.E. & ANDERSON, P. (1979). Misonidazole—a drug for trial in radiotherapy and oncology. *Int. J. Oncol. Biol. Phys.*, **5**, 851.

HAYNES, R.C. & LARNER, J.(1975). ACTH; adrenocortical steroids and their synthetic analogs; inhibitors of adrenocortical steriod biosynthesis. In: *The Pharmacological Basis of Therapeutics*. (Eds. Goodman & Gilman) Macmillan, New York, p. 1484.

JONES, D.H., BLEEHEN, N.M., WORKMAN, P. & SMITH, N.C. (1983). The role of microsomal enzyme inducers in the reduction of misonidazole neurotoxicity. *Br. J. Radiol.* **56.**

MOORE, S.L., PATERSON, I.C.M., NEWMAN, H. & VENABLES, S. (1981). Phenytoin-induced changes in the pharmacokinetics of misonidazole in radiotherapy patients. *Br. J. Cancer*, **44**, 592.

URTASUN, R.C., CHAPMAN, J.D., FELDSTEIN, M.L. & 6 others (1978). Peripheral neuropathy related to misonidazole: incidence and pathology. *Br. J. Cancer*, **37**, (suppl. III) 271.

URTASUN, R.C., TANASICHUK, H., FULTON, D. & 4 others (1982). High dose misonidazole with dexamethasone rescue: a possible approach to circumvent neurotoxicity. *Int. J. Radiat. Oncol. Biol. Phys.*, **8**, 365.

WASSERMAN, T.H., PHILLIPS, T.L., JOHNSON, R.J. & 6 others (1979). Intial United States clinical and pharmacological evaluation of misonidazole (Ro-07-0582), a hypoxic cell radiosensitizer. *Int. J. Radiat. Oncol. Biol. Phys.*, **5**, 775.

WASSERMAN, T.H., PHILLIPS, T.L., VAN RAALTI, G. & 6 others (1980). The neurotoxicity of misonidazole: potential modifying role of phenytoin sodium and dexamethazone (letter). *Br. J. Radiol*, **53**, 172.

WORKMAN, P. (1980*a*). Drug interactions with misonidazole: effects of dexamethasone and its derivatives on the pharmacokinetics and toxicity of misonidazole in mice. *Biochem. Pharmacol.*, **29**, 2769.

WORKMAN, P. (1980*b*). Dose-dependence and related studies in the pharmacokinetics of misonidazole and desmethylmisonidazole in mice. *Cancer Chemother. Pharmacol.*, **5**, 27.

WORKMAN, P., LITTLE, C.J., MARTEN, T.R. & 4 others (1978). Estimation of the hypoxic cell sensitiser misonidazole and its 0-demethylated metabolite in biological materials by reversed-phase high-performance liquid chromatography. *J. Chromatogr.*, **147**, 507.

WORKMAN, P., BLEEHEN, N.M. & WILTSHIRE, C.R. (1980). Phenytoin shortens the half life of the hypoxic cell radiosensitizer misonidazole in man: implication for possible reduced toxicity. *Br. J. Cancer*, **41**, 302.